# Molecular Hydrogen as a Potential Clinically Applicable Radioprotective Agent

**DOI:** 10.3390/ijms22094566

**Published:** 2021-04-27

**Authors:** Shin-ichi Hirano, Yusuke Ichikawa, Bunpei Sato, Haru Yamamoto, Yoshiyasu Takefuji, Fumitake Satoh

**Affiliations:** 1 Department of Research and Development, MiZ Company Limited, 2-19-15 Ofuna, Kamakura, Kanagawa 247-0056, Japan; y_ichikawa@e-miz.co.jp (Y.I.); b_sato@e-miz.co.jp (B.S.); info@e-miz.co.jp (F.S.); 2 Department of Molecular & Cell Biology, University of California, Berkeley, 3060 Valley Life Sciences Bldg #3140, Berkeley, CA 94720-3140, USA; haru.yamamoto@berkeley.edu; 3 Faculty of Environment and Information Studies, Keio University, 5322 Endo, Fujisawa 252-0882, Japan; takefuji@keio.jp

**Keywords:** molecular hydrogen, radiation-induced damage, medical application, radioprotective agent, non-DNA target, intracellular response, oxidation, inflammation, apoptosis, gene expression

## Abstract

Although ionizing radiation (radiation) is commonly used for medical diagnosis and cancer treatment, radiation-induced damages cannot be avoided. Such damages can be classified into direct and indirect damages, caused by the direct absorption of radiation energy into DNA and by free radicals, such as hydroxyl radicals (•OH), generated in the process of water radiolysis. More specifically, radiation damage concerns not only direct damages to DNA, but also secondary damages to non-DNA targets, because low-dose radiation damage is mainly caused by these indirect effects. Molecular hydrogen (H_2_) has the potential to be a radioprotective agent because it can selectively scavenge •OH, a reactive oxygen species with strong oxidizing power. Animal experiments and clinical trials have reported that H_2_ exhibits a highly safe radioprotective effect. This paper reviews previously reported radioprotective effects of H_2_ and discusses the mechanisms of H_2_, not only as an antioxidant, but also in intracellular responses including anti-inflammation, anti-apoptosis, and the regulation of gene expression. In doing so, we demonstrate the prospects of H_2_ as a novel and clinically applicable radioprotective agent.

## 1. Introduction

Ionizing radiation (radiation) is commonly used for medical diagnosis and cancer treatment. Amongst these uses, radiation therapy is known to be one of the most effective treatments for cancer. It is difficult to control radiation-induced damage with conventional radiation therapy; therefore, intensity-modulated radiation therapy (IMRT) has recently been used [1]. However, various radiation damages can also occur with IMRT. The harmful effects of radiation on the living body can be classified into direct and indirect effects. Direct effects are caused by the direct absorption of radiation energy into nucleic acids (DNA), proteins, and lipids [2,3,4,5]. Indirect effects are caused by free radicals, such as hydroxyl radicals (•OH), and molecular products generated in the process of water radiolysis [2,3,4,5]. In addition to the direct damage on DNA, secondary damages to non-DNA targets cannot be ignored because low-dose radiation damage is mainly caused by these indirect effects. Secondary damages include oxidation, inflammation, apoptosis, and effects on gene expression related to intracellular responses.

Medical applications of H_2_ were first reported by Dole et al. in 1975 [6]. They reported that the inhalation of hyperbaric H_2_ caused a marked regression in squamous cell carcinoma in mice induced by UV radiation. With the exception of a few studies, however, H_2_ has not been extensively studied for medical applications. In 2007, Ohsawa et al. reported that the inhalation of H_2_ gas ameliorated ischemia-reperfusion injury in a rat model with cerebral infarction [7]. In this paper, they showed that H_2_ is an antioxidant that selectively reduces highly oxidative reactive oxygen species (ROS) and reactive nitrogen species (RNS), such as •OH and peroxynitrite (ONOO^−^), respectively, but does not react with other ROS such as superoxide anions (O_2_^−^) and hydrogen peroxide (H_2_O_2_). However, we need to reacquaint ourselves with the pioneering paper on the antioxidant effects of H_2_ by Yanagihara et al. in 2005, two years before the study by Ohsawa et al. [8]. They reported that the ingestion of neutral H_2_-rich water produced by water electrolysis alleviated liver damage in rats induced by chemical oxidants. These papers have led to global research on the medical applications of H_2_. We recently showed that although H_2_ is an inactive substance, compared to other antioxidants, it is the only molecule with mitochondrial permeability and an ability to reduce •OH, which is promising for future medical applications [9,10]. Selective •OH scavengers may have potential medical applications as radioprotective agents. The efficacy of H_2_ against various diseases and disease models have been reported, and there are now more than 1000 papers on the medical applications of H_2_, including 80 clinical trials.

The use of a safer and more effective radioprotective agent in clinical practice is of great importance. Many drugs have been evaluated in a variety of ways. For instance, the radioprotective effects of many synthetic and natural compounds have been investigated. Cytokines such as granulocyte-macrophage colony-stimulating factor (GM-CSF), interleukin (IL)-1, IL-12, and natural compounds such as vitamin C, vitamin D, vitamin E, melatonin, succinate, alpha lipoic acid, and N-acetyl cysteine (NAC) have been reported to exhibit radioprotective effects in animal studies [11,12,13,14,15,16]. Many drugs are in various stages of evaluation, but many are far from being ideal radioprotective agents. However, amifostine (WR2721) has been developed as a radioprotective agent with free radical scavenging properties, such as against •OH, and is the only radioprotective agent approved by the U.S. FDA for clinical use [17,18,19,20,21,22]. However, this drug has not been widely considered as a useful radioprotective agent of choice because of its dose-dependent side effects such as hypotension, nausea, and vomiting [20]. Therefore, it is not an exaggeration to say that there are no clinically usable radioprotective agents with high efficacy and few side effects.

On the other hand, H_2_ has been reported to show radioprotective effects in many animal studies, and because H_2_ has also shown to have no side effects in clinical studies, it may be a clinically reliable radioprotective agent. As for its radioprotective effects in clinical trials, Kang et al. reported that H_2_-rich water improved the quality of life (QOL) of liver cancer patients receiving radiotherapy [23]. We recently reported that the inhalation of H_2_ gas reduced bone marrow damage in end-stage cancer patients receiving IMRT without compromising the antitumor effects [24,25]. This paper reviews previously reported radioprotective effects of H_2_ and discusses the mechanisms of H_2_ not only as an antioxidant, but also in intracellular responses including anti-inflammation, anti-apoptosis, and regulation of gene expression. In doing so, we demonstrate the prospects of H_2_ as a novel and clinically applicable radioprotective agent.

## 2. Biological Effects of Radiation

Exposure to radiation induces many detrimental effects, including genetic mutation, cell death, and carcinogenesis. The most radiation-sensitive organs are in the hematopoietic, digestive, reproductive, and skin systems, consisting of those with high cell proliferation [26,27]. Radiation damage occurs at the cellular level, either directly or indirectly. Thus, harmful effects of radiation on living organisms can be divided into direct and indirect effects [2,3,4,5].

**Figure 1:**
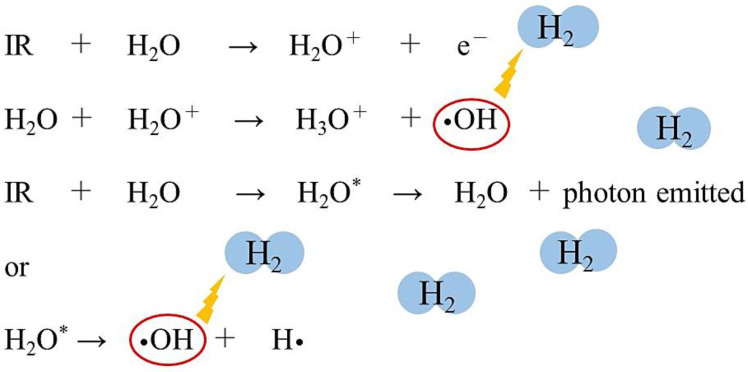
Ionizing radiation (IR) acts on water, a component of living organisms, ionizing and exciting the water molecules. Short-lived radical-cations (H_2_O^+^) are very unstable and decompose to produce hydroxyl radicals (•OH) and hydronium (H_3_O^+^). Electronically excited water molecules (H_2_O*) cleave to produce •OH and hydrogen radicals (H•). Molecular hydrogen (H_2_) can selectively eliminate the •OH by the following chemical reaction: •OH + H_2_ → H• + H_2_O.

Direct damages occur when radiation energy is directly absorbed by the target molecule, DNA. This direct action excites or ionizes the DNA, making it unstable because of the extra energy that is accumulated. In the process of releasing this extra energy, the ionization of DNA directly breaks chemical bonds in the DNA [2,3,4,5]. On the other hand, there are also indirect effects, which occur when molecules other than the target absorb radiation energy and produce active bodies, such as radicals, which eventually react with the target molecule. In aqueous solutions, radiation is first absorbed by water molecules to produce radicals and molecular products such as •OH, hydrogen radicals (H•), hydration electrons (e^−^_aq_), H_2_, and H_2_O_2_ [4] (Figure 1). These active substances then move through the water and induce chemical reactions with DNA.

In other words, radiation acts on water, which is a constituent of cells, and causes the ionization and excitation of water molecules. The water molecule ion (H_2_O^+^) is highly unstable and produces •OH and hydronium (H_3_O^+^). Excited water molecules (H_2_O*) cleave to produce •OH and H•. The electrons from the water molecules are trapped between other water molecules and produce e^−^_aq_ [4] (Figure 1). Approximately 60–70% of DNA damage is induced by the indirect action of free radicals [3].

The •OH produced during water radiolysis causes the oxidation of DNA, lipids, amino acids, and saccharides, and the oxidation of these biological materials leads to the formation of various secondary free radicals [26,27]. DNA is one of the major targets of free radicals. The compound 8-hydroxydeoxyguanosine (8-OHdG) is produced by •OH from deoxyguanosine in DNA and is considered to be one of the biomarkers of DNA damage and carcinogenesis [28,29]. Structural changes in proteins are induced by •OH and other free radicals, leading to functional changes in proteins [30]. Lipids in cell membranes are one of the major targets of •OH and other free radicals. Lipid peroxides such as malondialdehyde (MDA) and 2-thiobarbituric acid reactive substances (TBARS) are indicators of lipid damage [31]. These lipid peroxides induce changes in cell membrane permeability [32].

On the other hand, as an indirect effect of radiation, the molecular products generated by water radiolysis, such as e^−^_aq_, H_2_ and H_2_O_2_, also cause chemical reactions in biomolecules [4]. In particular, low doses of radiation induce modifications of intracellular molecules, leading to effects on oxidation, inflammation, apoptosis, and gene expression. It has been reported that there is also a bystander effect, in which information can be transmitted from exposed cells to unexposed cells, transferring radiation damage to these unexposed cells [33], as well as an abscopal effect, in which the local radiation therapy of a tumor can also shrink distant untreated tumors [34]. The involvement of radiation in cellular responses and the immune system has also been considered. Furthermore, the effects of radiation on epigenetic effects, i.e., changes in gene expression or cellular phenotypes that are inherited after cell division without changes in DNA sequence, have also been pointed out [35].

## 3. Radioprotective Effects of H_2_ in Animal Models

As for the radioprotective effects of H_2_ in animal models, protective effects on cognitive function, the immune system, lungs, heart, digestive organs, hematopoietic organs, testis, skin, and cartilage disorders have been reported [36,37,38,39,40,41,42,43,44,45,46,47,48,49,50,51,52,53]. An inhibitory effect on thymic lymphoma caused by radiation has also been reported [54]. The following is a summary of the literature that reports specific examples of the protective effects of H_2_ against different radiation disorders (Table 1).

### 3.1. Protective Effects on Cognitive Impairment

Liu et al. investigated the effect of H_2_-rich water (0.8–0.9 ppm) on radiation-induced cognitive dysfunction [36]. Rats were continuously administered with H_2_-rich water for 30 days before and after whole-brain irradiation using electron beams. Spatial recordings of the rats using the Morris water maze showed significant improvements in cognitive function in the H_2_-rich water group compared to the control group. In the H_2_-rich water group, the levels of superoxide dismutase (SOD), glutathione (GSH), and brain-derived neurotrophic factor (BDNF) in the brain were significantly higher, and the levels of MDA and 8-OHdG were significantly lower. In addition, mRNA and protein levels of BDNF and brain-derived neurotrophic factor receptor (TrkB) were also significantly higher in the H_2_-rich water group [36]. As such, they reported that the protective effect of H_2_ against radiation-induced cognitive dysfunction involves an antioxidant response, anti-inflammatory response, and protection of neonatal neurons by regulating the BDNF-TrkB signaling pathway.

### 3.2. Protective Effects on the Immune System

Radiation often causes the depletion of immune cells in tissues and blood, which leads to immunosuppression. Qian et al. reported the radioprotective effect of H_2_ on cultured human lymphocyte (AHH-1) cells [37]. Pretreatment with H_2_-rich PBS (1.2 ppm) prior to irradiation significantly reduced the levels of MDA and 8-OHdG in AHH-1 cells compared to untreated controls [37]. Furthermore, Qian et al. also reported the radioprotective effect of H_2_ in AHH1 cells in another paper. They showed that pretreatment with H_2_-rich saline (0.6 ppm) increased the viability of AHH-1 cells and inhibited apoptosis compared to non-treated cells [38].

Yang et al. also investigated the radioprotective effects of H_2_ on radiated AHH-1 cells, mouse thymocytes, and spleen cells [39]. Pretreatment with H_2_-rich medium (1.2 ppm) significantly reduced •OH in cultured AHH-1 cells compared to cells with no pretreatment. In addition, intraperitoneal administration of H_2_-rich saline (1.2 ppm) alleviated the apoptosis of thymocytes and splenocytes in mice, and inhibited the activation of caspase-3 compared to saline alone in mouse experiments. Moreover, H_2_-rich saline significantly ameliorated the depletion of white blood cells (WBCs) and platelets (PLT) in the peripheral blood of mice [39].

Zhao et al. reported the results of an experiment in which H_2_ protected against radiation-induced immune dysfunction [40]. They found that when H_2_-rich saline (1.2 ppm) was administered intraperitoneally prior to irradiation, H_2_ increased the spleen index calculated from mouse body weight and spleen weight and suppressed histopathological spleen damage. H_2_ also decreased ROS levels in spleen tissue, suppressed spleen apoptosis, and down-regulated pre-apoptotic proteins. In addition, H_2_ ameliorated radiation-induced T cell imbalance and regulated CD4^+^ T cell localization, Th-type cytokine, and pro-inflammatory cytokine levels. Based on these results, Zhao et al. reported that H_2_ has a radioprotective effect by scavenging ROS [40].

### 3.3. Protective Effects against Lung Injury

Terasaki et al. reported the protective effect of H_2_ against radiation-induced lung injury [41]. They irradiated A549 cells, a cell line of human lung epithelial cells, to induce radiation injury, and examined the effects of H_2_-rich PBS and H_2_-rich medium (both 1.2 ppm). In addition, mice were irradiated, and H_2_ gas (3%) inhalation or oral intake of H_2_-rich water (0.8 ppm) was used to reduce lung injury. H_2_ improved the survival rate of A549 cells, suppressed ROS production, and improved oxidative stress and apoptosis markers [41]. In the in vivo experiments using mice, H_2_ similarly attenuated oxidative stress and apoptotic markers, which were measured as acute lung injuries. H_2_ also alleviated chest computed tomography (CT), Ashcroft score (an index of lung fibrosis), and type III collagen deposition, which were each measured as indicators of chronic lung injury. They reported that H_2_ inhibited not only acute lung injury, but also chronic lung injury (lung fibrosis) [41]. A549 cells are tumor lines of lung adenocarcinoma; therefore, the fact that the survival rate of radiation-induced damaged cells was improved by H_2_ treatment may indicate that the antitumor effects of radiation may be compromised. However, because the rates of improvement in cell death by H_2_ is small compared to those by H_2_ for other oxidative stress markers, the attenuation of the antitumor effects may have little effect [41]. Indeed, in clinical trials on cancer patients receiving radiotherapy, Kang et al., as well as our own study, demonstrated that the antitumor effects by H_2_ were not compromised [23,24,25].

### 3.4. Protective Effects on Myocardial Injury

Qian et al. investigated the effects of pretreatment with H_2_-rich water (1.2 ppm) on radiation-induced myocardial damage in mice [42]. H_2_-rich water improved the survival rate and histopathological damage of the myocardium in mice compared to control groups. In addition, H_2_-rich water reduced myocardial MDA and 8-OHdG levels. Moreover, H_2_-rich water increased myocardial SOD and GSH levels compared to controls and alleviated myocardial cell DNA damage, as measured by the comet assay. Qian et al. reported that H_2_ has a cardioprotective effect against radiation-induced damage [42].

MicroRNAs (miRNAs) constitute a large class of post-transcriptional regulators of gene expression, and it is estimated that miRNAs regulate up to 30% of human protein-coding genes. They are implicated in many pathological processes, including radiation damage. Kura et al. investigated the involvement of miRNA-1, -15b, and -21 in the protective effects of H_2_-rich water (1.2 ppm) on rat myocardium damaged by radiation [43]. Radiation increased MDA and tumor necrosis factor (TNF-α) levels in myocardium, but H_2_ decreased these levels. miRNA-1, which is involved in myocardial hypertrophy, was decreased by irradiation, but H_2_ mitigated this decrease. miRNA-15b, which is involved in anti-fibrotic, anti-hypertrophic, and antioxidant effects, was decreased by radiation, but H_2_ reversed this effect. Furthermore, miRNA-21, which is involved in cardiac fibrosis, was increased by radiation, but H_2_ also reduced this increase. Based on these results, Kura et al. reported that the cardioprotective effects of H_2_ against radiation involves the regulation of miRNA-1, -15b, and -21 [43].

### 3.5. Protective Effects against Gastrointestinal Disorders

Qian et al. reported the radioprotective effects of H_2_ on cultured intestinal cells [37]. Pretreatment of human intestinal crypt cells (HIEC) with H_2_-rich PBS (1.2 ppm) prior to irradiation significantly inhibited apoptosis and increased the cell viability of HIEC cells compared to those with no pretreatment. They also examined the radioprotective effects of H_2_ in mice via the intraperitoneal administration of H_2_-rich saline before irradiation [38]. The results showed that, compared to the control group, the H_2_ group significantly alleviated the histopathological damage in the intestinal tract of mice, increased the levels of SOD and GSH in plasma, and significantly decreased the levels of MDA and 8-OHdG [38].

Xiao et al. reported the alleviating effects of H_2_-rich water (1.6 ppm) on gastrointestinal toxicity in a model produced by irradiating mice [44]. H_2_-water was administered orally by gavage before and after radiation. The results showed that H_2_-water significantly improved the survival rate and body weight of mice compared to the control, and furthermore improved the function of the intestine as seen from the gene expression of the intestinal epithelium. In a microarray analysis of the small intestine, H_2_-water down-regulated myeloid differentiation factor 88 (MyD88) expression. Furthermore, in high-throughput screening, H_2_-water improved the balance of intestinal bacteria impaired by radiation [44]. Xiao et al. reported that H_2_-water reduces radiation-induced gastrointestinal toxicity through the action of MyD88 on intestinal bacteria.

Qiu et al. investigated the radioprotective effects of H_2_ in animal experiments using mice and in cellular experiments using the intestinal crypt epithelial cell (IEC-6) line [45]. H_2_-rich saline (1.2 ppm) improved mouse survival and intestinal mucosal damage and function, as well as oxidative stress and inflammatory response. In vitro experiments using IEC-6 cells showed that H_2_ improved survival and inhibited ROS production. H_2_ inhibited mitochondrial depolarization, cytochrome c release, and the activities of caspase-3, caspase-9, and polymerase (PARP). In addition, H_2_ recovered from the decreased expression of B cell lymphoma-extra-large (Bcl-xl) and B cell lymphoma-2 (Bcl-2), proteins that suppress apoptosis, and suppressed the increased expression of BCL2-associated X protein (Bax), a protein that promotes apoptosis [45]. They suggested that the protective effects of H_2_ against radiation damage may involve the blockage of the mitochondrial apoptotic pathway.

### 3.6. Protective Effects against Hematopoietic Cell Injury

Zhang et al. reported the mitigating effects of H_2_-rich water on radiation-induced hematopoietic stem cell injury [46]. Mice were irradiated and orally administrated with H_2_-rich water (1.6 ppm) before and after the radiation. The results showed that H_2_ mitigated the injury of blood stem cells. In H_2_-treated c-kit^+^ cells, the mean fluorescence intensity of phosphorylated H2AX (γ-H2AX) and the percentage of 8-oxoguanine-positive cells were significantly decreased, suggesting that H_2_ alleviates radiation-induced DNA damage and oxidative DNA damage [46]. Furthermore, proteins related to the cell cycle, apoptosis, and oxidative stress were significantly ameliorated by H_2_ in irradiated mouse c-kit^+^ cells [46].

### 3.7. Protective Effects on Sperm Dysfunction

Chuai et al. reported the protective effects of H_2_ against radiation-induced impairment of spermatogenesis and hematopoiesis [47]. Intraperitoneal administration of H_2_-rich saline (1.2 ppm) prior to the radiation of mice significantly improved testicular sperm count and impaired spermatogenesis by histopathological analysis. In addition, the impairment of hematopoietic function by endogenous hematopoietic spleen colony formation (endoCFU), bone marrow nucleated cells (BMNC), and WBC in peripheral blood were significantly improved by pretreatment with H_2_-rich saline. They reported that H_2_-rich saline partially protected the spermatogenesis and hematopoietic functions of irradiated mice [47].

Chuai et al. also showed that in a cell-free system, •OH produced by the Fenton reaction and the radiolysis of water was reduced by H_2_ [48]. Furthermore, they found that the intraperitoneal administration of H_2_-rich saline (1.2 ppm) to mice prior to radiation significantly suppressed the reaction between •OH and intracellular macromolecules, indicating that radiation causes lipid peroxidation, protein carbonyls, and oxidative DNA damage [48]. In addition, Chuai et al. demonstrated the radioprotective effect of H_2_ on male germ cells from morphological changes in testicular tissue, apoptosis analysis and sperm quality test [48].

Jiang et al. reported the protective effects of H_2_-rich saline and amifostine (WR2721) against radiation-induced testicular damage in rats [49]. H_2_-rich saline (1.6 ppm) or WR2721 (200 mg/kg) was administered intraperitoneally before radiation. The results showed that testis weight, testis dimensions, sperm count, and sperm motility were all decreased by radiation in the control group, but significant improvement on these decreases was observed in the H_2_-rich saline and WR2721 groups. In addition, the control group showed a decrease in apoptotic index and SOD activity and an increase in MDA level, while H_2_-rich saline and WR2721 groups showed significant improvements in these parameters. Moreover, the H_2_-rich saline and WR2721 groups showed that the recovery of serum testosterone levels decreased with radiation [49].

### 3.8. Protective Effects against Skin Damage

The occurrence of dermatitis is a frequent side effect of radiotherapy in head and neck cancer patients. Therefore, Mei et al. examined the radioprotective effects of H_2_ (1.2 ppm) and its mechanism under local, single, and fractionated radiation conditions using human keratinocyte HaCaT cells and rats [50]. In experiments using HaCaT cells, the effects of H_2_ medium on cell viability, apoptosis, and biochemical assays were measured. The results showed that H_2_ significantly reduced the severity of dermatitis, accelerated tissue recovery, and inhibited weight loss in rats. H_2_ also showed protective effects when irradiated in three different increments. Moreover, H_2_ protected cells from radiation injury by improving the survival rate of HaCaT cells, inhibiting apoptosis, increasing SOD and GSH activities, and decreasing MDA levels [50]. Based on these results, they showed that H_2_ is useful in acute radiation-induced dermatitis.

Watanabe et al. examined the effects of prior inhalation of H_2_ gas (1.3%) on a radiation-induced skin injury model [51]. Inhalation of H_2_ significantly reduced the severity of radiation dermatitis and accelerated the repair of wounds with impaired healing. The percentage and staining levels of apoptotic keratinocytes in irradiated skin were examined by terminal deoxynucleotidyl transferase-mediated dUTP nick-end labeling (TUNEL) and 8-OHdG staining. These results were significantly lower in the H_2_-inhaled rats than in the non-H_2_-inhaled rats. In addition, the H_2_ inhalation group significantly reduced the delay in recovery of full-thickness skin wounds made at the site of X-ray irradiation [51]. These results suggest that prior inhalation of H_2_ may mitigate radiation-induced skin damage.

Zhou et al. investigated the effects of oral intake of H_2_-rich water (1.0 and 2.0 ppm) on a rat model with radiation-induced skin damage [52]. The H_2_ group significantly shortened healing times and increased healing rates of damaged skin, decreased the MDA and IL-6 levels, and increased the SOD activity and epidermal growth factor (EGF) content compared to the control group [52]. These results suggest that H_2_ promotes wound healing in radiation-induced skin lesions through its antioxidant and anti-inflammatory effects.

### 3.9. Protective Effects against Cartilage Damage

Although radiotherapy is a useful treatment for head and neck cancers, unexpected cartilage necrosis of the jaw often occurs as a radiation injury. Chen et al. investigated the protective effects of H_2_-rich saline (1.2 ppm) on a rat model with chondrocyte necrosis of the jaw induced by radiation in in vitro and in vivo experiments [53]. Treatment of bone marrow-derived mesenchymal stem cells (BMSCs) with H_2_ prior to irradiation significantly increased cell viability and differentiation potential and decreased ROS production compared to untreated controls. Rats in the control group showed an accumulation of myofibroblasts in and around the fibrotic medulla, but the accumulation was reduced in the H_2_ pre-treated group [53]. Chen et al. reported that the use of H_2_ against osteonecrosis of the jaw cartilage could be an important preventive and therapeutic strategy.

### 3.10. Inhibitory Effects on Carcinogenesis (Thymic Lymphoma)

Although radiation is a well-known carcinogen, the pathogenesis of radiation-induced thymic lymphoma is not well understood. Zhao et al. examined the protective effects of H_2_ on radiation-induced thymic lymphoma in mice [54]. The control group was irradiated for four weeks, and the H_2_ group was given H_2_-rich saline (1.2 ppm) intraperitoneally 5 min before each irradiation. As a result, the survival rate of mice, the incidence of lymphoma, the production of ROS in peripheral blood mononuclear cells (PBMC), and the levels of SOD, GSH, and MDA in plasma were all improved in the H_2_ group compared to the control group [54]. Zhao et al. reported that H_2_ protects against radiation-induced thymic lymphoma.

## 4. Radioprotective Effects of H_2_ in Humans

### 4.1. Improvement of Decreased QOL in Cancer Treatment

Cancer patients who have been irradiated often experience fatigue and decreased QOL. Radiation damage is attributed to radiation-induced oxidative stress and inflammation. Therefore, Kang et al. investigated the effects of H_2_-rich water on the improvement of QOL in patients with liver cancer who received radiation therapy [23]. The study was a randomized controlled trial with 49 patients. The placebo group (*n* = 24) ingested placebo water, and the H_2_ group (*n* = 25) ingested H_2_-rich water (1.2 ppm) for six weeks each.

The results revealed that the H_2_ group showed an improvement in the index related to oxidative stress compared to the placebo group. In addition, compared to the placebo group, the H_2_ group showed a significant improvement in QOL scores such as anorexia and taste disorder. Assuming that •OH is produced during and after irradiation and that H_2_ scavenges it, the antitumor effects of radiation may be impaired by H_2_. Therefore, Kang et al. investigated the effects of a placebo and H_2_ on tumor response. The results showed that the tumor responses of the placebo and H_2_ groups were similar, suggesting that the intake of H_2_-rich water did not impair the antitumor effects of radiation. They reported that H_2_-rich water improves the side effects of poor QOL without compromising the antitumor effects [23] (Table 1).

### 4.2. Improvement of Bone Marrow Damage in Cancer Treatment

Compared to conventional radiotherapy, IMRT has been developed to reduce side effects and is used clinically, but the reductions in side effects are insufficient. Therefore, we investigated the efficacy of H_2_ gas inhalation on bone marrow damage in end-stage cancer patients receiving IMRT [24,25]. The study was conducted as a retrospective observational study of 23 patients. Patients received IMRT for 1–4 weeks according to the irradiation protocol. Patients in the control group (*n* = 7) received 30 min of mild-pressure (1.35 atm) air inhalation in a chamber after each IMRT. On the other hand, patients in the H_2_ group (*n* = 16) also inhaled mild-pressure (1.35 atm) air and 5% H_2_ gas for 30 min in the chamber. The number of irradiations and total exposure doses of radiation in the control and H_2_ groups were almost the same. When bone marrow damage was compared before and after IMRT, the control group showed a significant decrease in WBC ratio and PLT ratio, while the H_2_ group significantly improved these decreases seen in the control group. Tumor response to IMRT in the control and H_2_ groups was similar, and the inhalation of H_2_ gas improved bone marrow damage without compromising the antitumor effects in cancer patients. Although this study examined the effects of mild-pressure H_2_ gas inhalation on radiation damage in cancer patients, we confirmed that the inhalation of H_2_ gas equivalent to mild-pressure H_2_ gas (1.35 times) in a normal pressure environment had the same radioprotective effects. Inhalation of H_2_ gas may be a new therapeutic strategy for bone marrow damage induced by IMRT [24,25] (Table 1).

## 5. Mechanism of the Radioprotective Effects of H_2_

As described in the previous section, there are both direct and indirect effects of radiation. Direct effects are damages to biomolecules such as DNA [2,3,4,5]. Indirect effects include oxidative damages caused by •OH, which is produced during water radiolysis, where •OH causes oxidation of various biological substances, and the oxidation of these biological substances leads to the generation of further secondary free radicals [2,3,4,5]. H_2_, on the other hand, is an inert substance, but it can protect living organisms from radiation-induced oxidative damage by selectively scavenging the large amounts of •OH generated in the living body. Although the radioprotective effects of H_2_ have been confirmed in the past literature, there are few that report the detailed mechanisms of H_2_ [36,37,38,39,40,41,42,43,44,45,46,47,48,49,50,51,52,53,54]. In this section, we will discuss the possible mechanisms of the radioprotective effects of H_2_ from these reports.

### 5.1. Antioxidant Effects

H_2_ selectively scavenges •OH, which is produced in large quantities during irradiation, and the scavenging of •OH can be considered as a direct effect of the radioprotective effects of H_2_. Chuai et al. showed that •OH is produced by the Fenton reaction and water radiolysis in cell-free systems, and it can be reduced by H_2_ [48]. Yang et al. [39], Zhang et al. [46] and Chuai et al. [48]. showed that H_2_ significantly reduces the •OH produced by radiation in in vitro and in vivo experiments. On the other hand, at the level of total ROS, Zhao et al. [40,54], Terasaki et al. [41], Qiu et al. [45] and Chen et al. [53] showed that H_2_ significantly reduces radiation-induced ROS production in in vitro and in vivo experiments, suggesting that the radioprotective effects of H_2_ involve the selective elimination of •OH by H_2_.

On the other hand, some studies have evaluated 8-OHdG as an indicator of DNA oxidation, MDA as an indicator of lipid oxidation, and both SOD and GSH activities as indicators of free radical scavenging systems to maintain the redox balance. Namely, the reduction in 8-OHdG and MDA levels by H_2_ has been reported by many authors [36,37,38,42,49,50,51,52,54]. In addition, the increase in SOD and GSH levels by H_2_ has been reported by many authors [37,40,42,49,50,52]. From these reports, we can assume that the radioprotective effect of H_2_ is largely due to the inhibition of oxidative stress.

We need to consider the mechanism of radioprotection by H_2_. •OH reacts non-specifically with many substances. The reaction rate of •OH with H_2_ in aqueous solution is much slower than with DNA, amino acids, sugars, and GSH [55]. However, Ohsawa et al. [7], Terasaki et al. [41] and Chuai et al. [48] reported that the amount of •OH in the medium produced by the Fenton reaction was reduced by H_2_, using electron spin resonance (ESR) methods. They also reported that the fluorescence of •OH was attenuated by H_2_ in an experiment using hydroxyphenyl fluorescein (HPF), a specific fluorescent dye for •OH [7,41,48]. Theoretically, for H_2_ to react with •OH, a higher concentration of H_2_ is required in the nucleus than for other solutes. Although future detailed studies are needed to resolve these contradictions, in aqueous solutions containing a large amount of solute, such as culture medium and buffer solutions, it may be necessary to consider factors, such as high intracellular diffusion rates of H_2_. It is also possible that the reaction rate of •OH and H_2_ is different in the nucleus.

If we assume that the only mechanism of the radioprotective effects of H_2_ is the selective elimination of •OH, the antitumor effects of radiation may be attenuated. However, in both Kang et al. and our reports of clinical trials examining radioprotective effects in cancer patients, H_2_ did not attenuate the antitumor effects by radiation [23]. Kang et al. showed that H_2_ improved the oxidative stress-related index, suggesting that the radioprotective effects of H_2_ may be due to its antioxidant effect, but that other biological defense systems, including hormones and enzymes involved in radiation protection, may also be at work [23]. We also reported that the radioprotective effects of H_2_ may involve not only the direct scavenging of •OH, but also indirect effects through the activation of host-mediated antioxidant and anti-inflammatory systems [24,25]. The possibility that the radioprotective effects by H_2_ involves an indirect effect, rather than a direct effect, on •OH is supported by the study schedule in which patients inhaled H_2_ gas after IMRT, but not before.

### 5.2. Anti-Inflammatory Effects

Chronic inflammation caused by radiation exposure is closely related to oxidative damage. Yahyapour et al. reported in their review that the long-term effects of radiation exposure accidents include increased risk of cancer, but also many inflammation-related diseases and autoimmune diseases [56]. They reported that cytokines including IL-1, TNF-α and interferon-γ (IFN-γ) play an important role as indicators of chronic inflammatory damage and oxidative damage after radiation exposure [56]. Indeed, in a report by Kura et al. that examined the protective effect of H_2_ on a rat model of myocardial injury induced by irradiation, H_2_ significantly reduced MDA and TNF-α levels in the myocardium [43]. Zhou et al., who examined the radioprotective effects of H_2_ on a rat skin damage model, showed that H_2_ significantly reduced MDA and IL-6 levels in the damaged skin [52]. In a recent review, we reported that •OH generated in mitochondria induces oxidative stress in mitochondrial DNA (mtDNA), and that oxidized mtDNA triggers a cascade of inflammatory cytokine release from nucleotide-binding and oligomerization domain-like receptor family pyrin domain-containing 3 (NLRP3) to IL-1 and IL-18 [57]. The mechanism of H_2_-induced amelioration of chronic inflammatory diseases may involve the scavenging of •OH generated in mitochondria [57].

### 5.3. Anti-Apoptotic Effects

Apoptosis, or cell death caused by radiation, is also closely related to oxidative damage and inflammation. It has been reported in the literature that H_2_ has a radioprotective effect on radiation-induced cell or animal models through its anti-apoptotic effects [37,39,40,41,45,48,49,50,51]. The TUNEL assay and the quantification of caspases (caspase-3, caspase-8, and caspase-9), which are essential proteases for apoptosis, have been used to evaluate the anti-apoptotic effects of H_2_ on radiation injury models. It can also be assessed by examining the expression of Bcl-xL and Bcl-2, proteins that inhibit apoptosis, and Bax, a protein that induces apoptosis. For example, Watanabe et al. measured the percentage and staining level of apoptotic keratinocytes in irradiated skin by TUNEL and 8-OHdG staining in an experiment to evaluate the efficacy of H_2_ on a radiation-induced skin damage model and showed that these were reduced by H_2_ [51]. In addition, in cell experiments using IEC-6, an intestinal crypt epithelial cell line, Qiu et al. showed that H_2_ inhibits mitochondrial depolarization, cytochrome c release, and the activities of caspase-3, caspase-9, and PARP [45]. They further reported that H_2_ exerts an anti-apoptotic effect by recovering from the decreased expression of Bcl-xl and Bcl-2 and inhibiting the increased expression of Bax [45].

### 5.4. Regulation of Gene Expression

**Figure 2:**
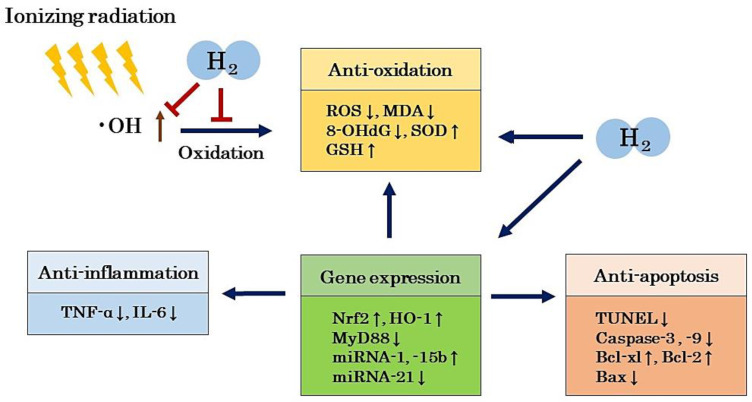
H_2_ not only has a direct radioprotective effect by scavenging •OH, but also indirectly by regulating gene expression, exhibiting antioxidant, anti-inflammatory, and anti-apoptotic effects, which may lead to radioprotective effects. H_2_: molecular hydrogen; •OH: hydroxy radical; ROS: reactive oxygen species; GSH: glutathione; TNF-α: tumor necrosis factor-α; IL-6: interleukin-6; Nrf2: nuclear factor erythroid 2-related factor; HO-1: heme oxygenase-1; TUNEL: terminal deoxynucleotidyl transferase-mediated dUTP nick-end labeling; Bcl-xl: B-cell lymphoma-extra-large; Bcl-2: B-cell lymphoma-2; Bax: BCL2-associated X protein; MyD88: myeloid differentiation factor 88; miRNA: microRNA.

Nuclear factor erythroid 2-related factor (Nrf2), an endogenous antioxidant regulator, is closely correlated with the enhancement of SOD and catalase (CAT). In addition, Nrf2 has biological protective effects such as enhancing heme oxygenase-1 (HO-1) activity, which exhibits cytoprotective effects such as anti-inflammation and antioxidation. Many studies have reported that H_2_ promotes the expression of Nrf2 and bioprotective responses through HO-1 and other bioprotective proteins [58,59,60]. Xiao et al. examined the mitigating effects of H_2_ on gastrointestinal disorders in a model created by irradiating mice [44]. They reported that H_2_ down-regulated MyD88 expression in a microarray analysis of the small intestine [44]. Furthermore, Kura et al. reported experimental results showing that H_2_ regulates the expression of miRNAs involved in myocardial oxidation, hypertrophy, or fibrosis in a rat model with myocardial injury induced by radiation [43]. These results suggest that H_2_ not only has a direct radioprotective effect by scavenging •OH, but also indirect effects by regulating gene expression and exhibiting antioxidant, anti-inflammatory, and anti-apoptotic effects (Figure 2).

## 6. Prospects of H_2_ as a Radioprotective Agent

Radiation damage occurs during radiotherapy for cancer patients and during medical diagnostic procedures such as CT, even though the amount of radiation exposure is small. Therefore, attempts are being made to develop radioprotective agents that are safe and effective; however, the only radioprotective agent currently accepted in clinical use is amifostine [17,18,19,20,21,22]. This drug is used to protect normal tissues around tumors from radiation damage during radiotherapy. Amifostine is rapidly taken up by normal tissues, but its uptake into the tumor is slow. Therefore, irradiation within a few minutes after amifostine administration is thought to selectively protect normal tissues [20]. The mechanism for the radioprotective effects of amifostine includes the scavenging of free radicals, such as •OH, produced during irradiation [22]. However, amifostine is not considered as a useful radioprotective agent because of various safety issues and dose-dependent side effects such as hypotension, nausea, and vomiting [20]. On the other hand, edaravone, a •OH scavenger, has been used clinically as a treatment for acute cerebral infarction [61]. Based on the mechanisms of this drug, basic studies on its radioprotective effects have been conducted using cultured cells and experimental animals [62], but it has not yet been used clinically as a radioprotective agent. In addition, antioxidants such as vitamin C, vitamin D, vitamin E, and melatonin have also been studied for their potential as radioprotective agents [11,16,20]. However, they are not as safe as H_2_, because it has been reported that the overdose of these antioxidants increases mortality in systematic reviews and meta-analyses [63].

On the other hand, H_2_ is a novel antioxidant that exhibits antioxidant, anti-inflammatory, anti-apoptotic, and anti-allergic effects. H_2_ has both preventive and therapeutic effects on a wide variety of diseases including cancer [64], sepsis [65], cardiovascular disease [66], brain and nerve diseases [67], diabetes [68], and metabolic syndrome [69]. H_2_ can be used in a variety of ways, including inhalation as H_2_ gas, consumption as H_2_-rich water, and intravenous administration as H_2_-dissolved saline, etc. Each of these administration methods has its own characteristics, but H_2_ gas inhalation provides the highest amount of H_2_ in a time-dependent manner. This can be explained by the fact that the maximum blood and tissue concentrations (Cmax) in H_2_ gas inhalation are lower, while their area under the curve (AUC) is extremely high compared to those of other administration routes [70,71]. We believe that the inhalation of H_2_ is suitable for use as a radioprotective agent. In addition, H_2_ has no safety issues, because no side effects have been observed in clinical trials [10,64,66,69,72,73,74,75,76,77] and from the fact that H_2_ does not affect normal tissues and cells [78].

In our large intestine, H_2_ is produced by H_2_-producing bacteria [79,80], and the amount produced depends on our diet and lifestyle [81]. However, most H_2_ is not used in our body and is discharged via exhalation or flatulence [82]. When H_2_ is consumed in the form of H_2_ water, it diffuses from the walls of the stomach and intestines to the surrounding organs and tissues, while also travelling throughout the body via the bloodstream [70,71]. Similarly, when H_2_ is inhaled as H_2_ gas, it diffuses from the lung tissues to the surrounding organs and tissues, and is distributed throughout the body via the blood [70,71]. H_2_ is a very small molecule; therefore, it has physical properties that allow it to easily pass through the cell membrane and diffuse into the cytoplasm. In a short amount of time, H_2_ is able to reach the mitochondria and nucleus to protect them [7]. H_2_ can also easily cross the blood–brain barrier.

In terms of H_2_ as a radioprotective agent, H_2_ has shown excellent efficacy in irradiated cells and animal models [36,37,38,39,40,41,42,43,44,45,46,47,48,49,50,51,52,53,54], improved the QOL of liver cancer patients treated with radiation therapy, and reduced bone marrow damage in end-stage cancer patients treated with IMRT [23,24,25]. Amifostine has been used clinically as a free radical scavenger and edaravone, another scavenger, has also been investigated as a potential radioprotective agent [17,18,19,20,21,22,61], although both lack efficacy and safety compared to H_2_. Based on the properties above, H_2_ may be an ideal radioprotective agent with clinical applicability.

A possible mechanism for the radioprotective effects of H_2_ is the direct elimination of •OH. However, it is also necessary to consider the indirect mechanisms of •OH. In cellular and animal models in which radioprotective effects were examined, H_2_ showed not only antioxidant but also anti-inflammatory, anti-apoptotic, and gene expression regulating effects [36,37,38,39,40,41,42,43,44,45,46,47,48,49,50,51,52,53,54]. It is possible that H_2_ may also indirectly exert its antioxidant effects by regulating gene expression through intracellular responses. The direct effect of H_2_ on radioprotection, or the elimination of •OH, is well explained, but the indirect effects remain unclear and require further study.

## 7. Conclusions

In this review, we reported the radioprotective effects of H_2_ in cellular and animal models on cognitive function, immune system, lung, heart, digestive organs, hematopoietic organs, testis, skin, and cartilage damages [36,37,38,39,40,41,42,43,44,45,46,47,48,49,50,51,52,53]. We also reported the inhibitory effect of H_2_ on thymic lymphoma induced by radiation [54]. As for radioprotective effects in clinical trials, improvement of QOL with H_2_ in patients with liver cancer receiving radiotherapy was reported by Kang et al. [23]. We also recently reported that the inhalation of H_2_ gas reduced bone marrow damage in end-stage cancer patients receiving IMRT [24,25]. The mechanism of the radioprotective effects of H_2_ is thought to be related to the scavenging of free radicals such as •OH, but it is also suggested to be indirectly related to anti-inflammatory and anti-apoptotic characteristics, as well as the regulation of gene expression of intracellular signaling. H_2_ has been widely applied clinically in areas other than radiation damage and has been reported to be a substance with excellent efficacy and safety. Therefore, H_2_ may have potential clinical applications as a radioprotective agent, and could be used therapeutically against radiation damage in the future.

## Figures and Tables

**Table 1:** Radioprotective effects of H_2_ in cell free system, cells, animal models and clinical trials.

Damages/Damage Models	Species/Cells	Effects of H_2_	Ref. No.
Cell-free system		•OH is produced by the Fenton reaction and water radiolysis, and it was reduced by H_2_.	[48]
Cognitive impairment	Rats	Radiation-induced cognitive dysfunction was protected by H_2_-rich water.	[36]
Immune dysfunction	AHH-1 cells	Pretreatment with H_2_-rich PBS prior to radiation reduced the levels of MDA and 8-OHdG.	[37]
	AHH-1 cells	Pretreatment with H_2_-rich saline increased the viability of AHH-1 cells and inhibited apoptosis.	[38]
	AHH-1 cells	Pretreatment with H_2_-rich medium reduced •OH induced by radiation.	[39]
	Mice	H_2_-rich saline protected immunocytes from radiation-induced apoptosis.	[39]
	Mice	H_2_-rich saline protected against radiation-induced immune dysfunction.	[40]
Lung damage	A549 cells	H_2_-rich PBS suppressed ROS production, and improved oxidative stress and apoptosis markers.	[41]
	Mice	H_2_ gas inhibited not only acute lung damage, but also chronic lung damage.	[41]
Myocardial damage	Mice	H_2_-rich water protected against radiation-induced myocardium damage.	[42]
	Rats	H_2_-rich water protected against radiation-induced myocardium damage.	[43]
Gastrointestinal damage	HIEC	H_2_-rich PBS inhibited apoptosis and increased the cell viability of HIEC.	[37]
	Mice	H_2_-rich saline protected against radiation-induced gastrointestinal disorders.	[38]
	Mice	H_2_ water ameliorated radiation-induced gastrointestinal toxicity.	[44]
	IEC-6 cells	H_2_-rich medium improved survival and inhibited ROS production.	[45]
	Mice	H_2_-rich saline improved mouse survival and intestinal mucosal damage and function.	[45]
Hematopoietic cell injury	Mice	H_2_-rich water ameliorated radiation-induced hematopoietic stem cell injury.	[46]
Spermatogenesis and hematopoiesis disorders	Mice	H_2_-rich saline protected spermatogenesis and hematopoietic functions of irradiated mice.	[47]
Testicular damage	Rats	H_2_-rich saline protected against radiation-induced testicular damage.	[49]
Skin damage	HaCaT cells	H_2_-rich medium protected HaCaT cells from radiation injury by improving the survival rate.	[50]
	Rats	H_2_-rich saline reduced the severity of dermatitis, accelerated tissue recovery, and inhibited weight loss.	[50]
	Rats	Prior inhalation of H_2_ gas mitigated radiation-induced skin damage.	[51]
	Rats	H_2_-rich water promoted wound healing in radiation-induced skin lesions.	[52]
Cartilage damage	BMSC	H_2_-rich medium increased cell viability and differentiation potential.	[53]
	Rats	H_2_-rich saline protected against the osteonecrosis of jaw cartilage induced by radiation.	[53]
Thymic lymphoma	Mice	H_2_-rich saline protected against radiation-induced thymic lymphoma.	[54]
Impaired QOL	Humans	H_2_-rich water improved side effects of poor QOL by radiation therapy.	[23]
Bone marrow damage	Humans	H_2_ gas inhalation protected bone marrow damage in cancer patients receiving IMRT.	[24,25]

H_2_: molecular hydrogen; •OH: hydroxy radical; AHH-1: human lymphocyte cell; MDA: malondialdehyde; 8-OHdG: 8-hydroxydeoxyguanosine; ROS: reactive oxygen species; HIEC: human intestinal crypt cell; IEC-6: intestinal crypt epithelial cell; HaCaT: human keratinocyte cell; BMSC: marrow-derived mesenchymal stem cell; QOL: quality of life; IMRT: intensity-modulated radiation therapy; Ref.: references.

## Data Availability

The data presented in this study are available on request from the corresponding author.
